# Closed Genome Sequence of Clostridium pasteurianum ATCC 6013

**DOI:** 10.1128/genomeA.01596-14

**Published:** 2015-02-19

**Authors:** Carlo Rotta, Anja Poehlein, Katrin Schwarz, Peter McClure, Rolf Daniel, Nigel P. Minton

**Affiliations:** a The Clostridia Research Group, BBSRC/EPSRC Synthetic Biology Research Centre, School of Life Sciences, Centre for Biomolecular Sciences, University of Nottingham, Nottingham, United Kingdom; b Genomic and Applied Microbiology & Göttingen Genomics Laboratory, Georg-August-University Göttingen, Göttingen, Germany; c Unilever, Research and Development, Bedford, United Kingdom

## Abstract

We report here the closed genome of Clostridium pasteurianum ATCC 6013, a saccharolytic, nitrogen-fixing, and spore-forming Gram-positive obligate anaerobe. The organism is of biotechnological interest due to the production of solvents (butanol and 1,3-propanediol) but can be associated with food spoilage. The genome comprises a total of 4,351,223 bp.

## GENOME ANNOUNCEMENT

Clostridium pasteurianum ATCC 6013 is a saccharolytic and spore-forming, Gram-positive obligate anaerobe. Able to fix atmospheric nitrogen, it can grow on glycerol to produce the commercial solvents butanol and 1,3-propanediol (1, 2). Its ability to produce heat-resistant spores and grow at a low pH mean it is also linked with food spoilage, particularly of canned vegetables (3, 4).

C. pasteurianum ATCC 6013 was obtained from the American Type Culture Collection. TheMasterPure complete DNA purification kit (Epicentre, Madison, WI, USA) was used to isolate genomic DNA. For whole-genome sequencing, a combined approach employing a 454 GS-FLX system (Titanium GS70 chemistry, Roche Life Science, Mannheim, Germany) and an Illumina MiSeq benchtop sequencer (Deepseq, University of Nottingham) was used. Preparation of paired-end and shotgun libraries (only 454) as well as sequencing were performed as described by the manufacturers. Sequencing resulted in 102,095,674 paired-end Illumina reads, 690,412 454 shotgun reads, and 493,571 454 paired-end reads. The initial hybrid *de novo* assembly was performed with the Roche Newbler assembly software 2.9 for scaffolding and the MIRA software (5) and resulted in 17 scaffolds comprising 93 contigs. For gap closure, the Gap4 (v.4.11) software of the Staden package (6) was used. Gaps were closed by PCR-based techniques and Sanger sequencing of the products employing BigDye 3.0 chemistry and an ABI3730XL capillary sequencer (Applied Biosystems, Life Technologies GmbH, Darmstadt, Germany). The complete genome comprises one circular chromosome of 4,351,223 bp with an overall GC content of 30%. The software tool prodigal (Prokaryotic Dynamic Programming Gene finding Algorithm) (7) was used for automatic gene prediction, and the identification rRNA and tRNA genes was performed with RNAmmer (8) and tRNAscan (9), respectively. The IMG/ER (Integrated Microbial Genomes/Expert Review) system (10, 11) was used for automatic annotation, which was subsequently manually curated using the Swiss-Prot, TREMBL, and InterPro databases (12).

The genome harbors 10 rRNA operons, 81 tRNA genes, 3,220 predicted protein-encoding genes with function prediction, and 768 putative genes coding for hypothetical proteins. Present are a number of type II restriction/methylation proteins that act as a barrier to DNA transfer, including *bepIM*, previously identified as CpaAI (13), *dpnB*, a DpnII-type system previously identified as *CpaI* (14), and a single type I system (*hsdRMS*)*.* Those genes expected to be present in a saccharolytic species (15–17) producing acetate (*ackA* and *pta*) and butyrate (*buk* and *ptb*), as well as the genes (*thl, hbd*, *crt*, *etfAB*, and *bcd*) encoding the enzymes responsible for the conversion of acetyl-CoA to butyryl-CoA, and those responsible for 1,3-propanediol (glycerol dehydratase and 1,3-propandiol dehydrogenase) and butanol production (AdhE, alcohol/aldehyde dehydrogenase) were also noted. Present are eight GerK spore germination receptors, with 3 orphan *gerKB* genes and a *gerKA-gerKC* and a *gerKA*-*gerKC*-*gerKB* cluster, and two copies of a spore cortex-lytic SleB enzyme (18). The Spo0A master regulator of sporulation is atypical. It carries a lysine residue at position 255 as opposed to the glutamine present in other clostridial Spo0A proteins (19), including that of C. pasteurianum DSM525. Strain ATCC 6013 produces higher spore titers than DSM 525 (20).

### Nucleotide sequence accession number.

The genome sequence has been deposited in GenBank under the accession number CP009267.
